# In Vivo Observation of Cutaneous Larva Migrans by Fluorescence-Advanced Videodermatoscopy

**DOI:** 10.3201/eid2701.203137

**Published:** 2021-01

**Authors:** Alice Ramondetta, Simone Ribero, Pietro Quaglino, Paolo Broganelli

**Affiliations:** Università degli Studi di Torino, Turin, Italy

**Keywords:** in vivo observation, cutaneous larva migrans, hookworms, fluorescence-advanced videodermatoscopy, diagnostic technique, creeping eruption, parasites

## Abstract

Fluorescence-advanced videodermatoscopy is not a widespread diagnostic technique. Its application in dermatology can facilitate the diagnosis of diseases such as cutaneous larva migrans by enabling us to recognize the precise position of larva in vivo on the skin. Using this noninvasive technique, we detected a case of cutaneous larva migrans in a patient.

Dermoscopy alone is not useful for identifying cases of cutaneous larva migrans. Therefore, we conducted a study to introduce a simple and useful noninvasive method for clinical practice. Although still relatively unknown, this method provides more information than clinical procedures and simple dermatoscopy.

## The Study

We report the case of a 34-year-old man who came to our attention at the Dermatological Clinic of the University of Turin (Turin, Italy) after the appearance of an intensely itchy, erythematous-papular serpiginous lesion that was gradually increasing in size. It was located near the right groin. Our suspicion that it was cutaneous larva migrans was validated because the patient had returned from Thailand, a country to which this infestation is endemic, where he had gone for a seaside holiday.

Dermoscopy alone was not useful for identifying the cause in this case. Although the diagnosis of larva migrans is usually clinical, to obtain a diagnosis of certainty by using a biopsy specimen of suspected larva, we used fluorescence-advanced videodermatoscopy to identify its precise position (*1*).

Fluorescence-advanced videodermatoscopy is an optical electronic system that uses a monochromatic light-emitting source with an a mean ± SD λ of 405 ± 5 nm and a field of view of 340 μm to examine the skin. This system uses the ability of endogenous molecules to absorb specific wavelengths and emit fluorescence. The examination is conducted in vivo, and the optical device is directly applied to the skin by using liquid paraffin oil as an interface. The images are visualized in real time by using greyscale to indicate the levels of light absorption (i.e., black indicates no fluorescence and white indicates the highest fluorescence) (*2*).

In this instance, by positioning the probe on the surrounding skin downstream of the distal end of the serpiginous path, within an area of 0.5 cm, we were able to recognize an oval-shaped figure with a rounded tip, a darker gray color than the adjacent tissue (undamaged skin), and a white linear outline of the larva. (Figure, panel A). Once the actual position of the larva was recognized, a 4-mm punch biopsy specimen was obtained and analyzed microscopically; we obtained subsequent histologic confirmation. (Figure, panel B).

**Figure:**
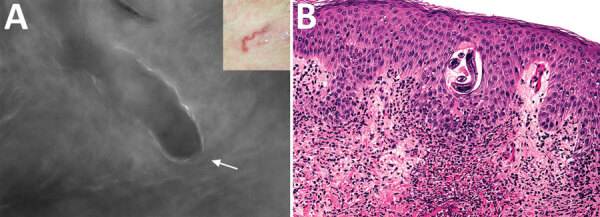
Imaging and biopsy results for patient with cutaneous larva migrans, Turin, Italy. A) Fluorescence-advanced videodermatoscopy showed larva with a diameter of 70–80 μm, located intraepidermally, ≈0.5 cm to the right of the distal end of the serpentine path (indicated in the inset) caused by its passage in the skin. White arrow indicates head of the larva (original magnification ×500). B) Hematoxylin and eosin–stained longitudinal skin section obtained from a 4-mm biopsy specimen, showing the cavity at the epidermal level in which it is possible to observe the larva inside (original magnification ×20). Once the biopsy specimen was obtained, confirmation of a larva by morphologic details or molecular techniques was necessary to differentiate animal nematodes from other larvae, particularly *Strongyloides stercoralis*, because treatment and follow-up would be different.

We prescribed a 7-day therapy regimen with topical ivermectin, under occlusion. Complete resolution of the cutaneous lesion was obtained (*3*). It was not essential to perform a biopsy for successful treatment; however, although oral ivermectin is the most recommended treatment, because of its side effects and the difficulty in obtaining this drug, the topical formulation is preferable and is equally effective as other localized forms of this drug.

## Conclusions

Cutaneous larva migrans (creeping eruption) is a cutaneous disease that manifests as an erythematous migrating linear or serpiginous tract because of penetration of a hookworm larva into the epidermis. The hookworms most frequently responsible for cutaneous larva migrans are the dog hookworms *Ancylostoma braziliense* or *An. caninum.* These hookworms are found worldwide, predominantly in tropical and subtropical countries, such as those in Southeast Asia, Africa, South America, the Caribbean, Australia, and southeastern parts of the United States. A similar condition known as larva currens is caused by *Strongyloides stercoralis* roundworms and should be considered in the differential diagnosis.

The life cycle of these nematodes includes male and female adult stages in the intestines of dogs and cats; eggs are passed in the feces of the host and are deposited in the soil (sandy beaches, sand boxes, under dwellings). Under favorable conditions, these eggs hatch and give rise to the infective larval phase. Humans, defined as dead-end hosts, might become infected when infective filariform larvae in soil penetrate the skin. Within a few days, an increased erythematous or vesiculobullous serpiginous track will appear, accompanied by intense pruritis at the site of larval penetration. Larvae migrate at a rate of several millimeters per day (*4*), and lesions are ≈3 mm wide and might be up to 15–20 mm in length. The larva is usually located 1–2 cm ahead of the eruption (*5*). Vesiculobullous lesions develop in ≈10% of cases (*6*). In comparison, the rash of larva currens is typically pink, evanescent, and urticarial and might be linear, serpiginous, annular, arcuate, or plaque-like (*7*). This rash usually appears on the buttocks and abdomen during the chronic autoinfective stage of strongyloidiasis.

The diagnosis of cutaneous larva migrans is based on clinical history and physical findings. Infected patients typically have a history of exposure to contaminated soil or sand (walking barefoot or lying on sand) and the characteristic serpiginous lesion on the skin. Nevertheless, a definitive diagnosis by biopsy specimen sampling is difficult to obtain because the precise position of the larva is unpredictable. To overcome this problem, flight theory was applied by identification of an uncertainty circle in which the possible position of the larva would be obtained by means of a mathematical formula. The distance D between the last observation point and the possible actual point would be obtained by multiplying 3 parameters: D = V × T × R, in which V is the speed of the larva (mm/d), T is the time elapsed between the last and possible actual observations, and R is a multiplying factor that takes into account the characteristics of the path (*8*).

It would be useful to have a procedure or instrument that can identify in vivo the precise position of the larva to enable accurate biopsy sampling. Methods to identify the position of the larva downstream from the distal end of the serpiginous tract have used dermoscopy; features identified, including translucent, brown, structureless areas corresponding to larval bodies and red-dotted vessels corresponding to an empty burrow, have been reported (*9*).

In conclusion, fluorescence-advanced videodermatoscopy, a simple-to-use method of noninvasive diagnosis, is not a widely used procedure. However, our results show that it appears useful for examining of skin that is apparently healthy or does not have specific clinical–dermoscopic parameters, especially in the context of parasitology, which enables immediate recognition of the etiologic agent (*10*).
